# A Modified Glycosaminoglycan, GM-0111, Inhibits Molecular Signaling Involved in Periodontitis

**DOI:** 10.1371/journal.pone.0157310

**Published:** 2016-06-16

**Authors:** Justin R. Savage, Abigail Pulsipher, Narayanam V. Rao, Thomas P. Kennedy, Glenn D. Prestwich, Maria E. Ryan, Won Yong Lee

**Affiliations:** 1 GlycoMira Therapeutics, Inc. Salt Lake City, UT, 84108, United States of America; 2 Pulmonary Diseases Critical Care and Environmental Medicine, School of Medicine, Tulane University, New Orleans, LA, 70112, United States of America; 3 Department of Medicinal Chemistry and Center for Therapeutic Biomaterials, University of Utah, Salt Lake City, UT, 84108, United States of America; 4 Department of Oral Biology and Pathology, School of Dental Medicine, Stony Brook University, Stony Brook, NY, 11794, United States of America; Université de Lyon - Université Jean Monnet, FRANCE

## Abstract

**Background:**

Periodontitis is characterized by microbial infection, inflammation, tissue breakdown, and accelerated loss of alveolar bone matrix. Treatment targeting these multiple stages of the disease provides ways to treat or prevent periodontitis. Certain glycosaminoglycans (GAGs) block multiple inflammatory mediators as well as suppress bacterial growth, suggesting that these GAGs may be exploited as a therapeutic for periodontitis.

**Methods:**

We investigated the effects of a synthetic GAG, GM-0111, on various molecular events associated with periodontitis: growth of *Porphyromonas gingivalis* (*P*. *gingivalis*) and *Aggregatibacter actinomycetemcomitans* (*A*. *actinomycetemcomitans*) pathogenic bacteria associated with periodontitis; activation of pro-inflammatory signaling through TLR2 and TLR4 in mouse macrophage RAW 264.7 cells and heterologously expressed HEK 293 cells; osteoclast formation and bone matrix resorption in cultured mouse pre-osteoclasts.

**Results:**

(1) GM-0111 suppressed the growth of *P*. *gingivalis* and *A*. *actinomycetemcomitans* even at 1% (w/v) solution. The antibacterial effects of GM-0111 were stronger than hyaluronic acid (HA) or xylitol in *P*. *gingivalis* at all concentrations and comparable to xylitol in *A*. *actinomycetemcomitans* at ≥2% (w/v) solution. We also observed that GM-0111 suppressed biofilm formation of *P*. *gingivalis* and these effects were much stronger than HA. (2) GM-0111 inhibited TLR-mediated pro-inflammatory cellular signaling both in macrophage and HEK 293 cells with higher selectivity for TLR2 than TLR4 (IC_50_ of 1–10 ng/mL *vs*. > 100 μg/mL, respectively). (3) GM-0111 blocked RANKL-induced osteoclast formation (as low as 300 ng/mL) and bone matrix resorption. While GM-0111 showed high affinity binding to RANKL, it did not interfere with RANKL/RANK/NF-κB signaling, suggesting that GM-0111 inhibits osteoclast formation by a RANKL-RANK-independent mechanism.

**Conclusions:**

We report that GM-0111 inhibits multiple molecular events involved in periodontitis, spanning from the early pro-inflammatory TLR signaling, to pathways activated at the later stage component of bone loss.

## Introduction

Periodontitis is a chronic inflammatory disease characterized by recurrent infection and inflammation that often progresses into alveolar bone loss. Moderate to severe forms of periodontitis occur in approximately 10%-15% of middle-aged adults worldwide and approximately 20% of US adults [1–3]. Currently available treatment options are limited. There is a need for more effective treatments to control this common disease.

In healthy individuals, the periodontium maintains local tissue homeostasis by balancing its immune response to the local microbial ecosystem. Diseases or drastic alterations of the local microenvironment in the oral cavity can break this homeostasis, leading to dysbiotic microbial ecosystems in the periodontium. This process in turn increases the host immune responses, causes tissue destruction, enhances the proliferation of pathogenic microorganisms, and further exacerbates the host immune responses [4,5]. The development of this vicious cycle is key to the pathogenesis of periodontitis. Therapeutics for periodontitis should aim to break this continuum and restore tissue homeostasis in the periodontium.

Considerable research has focused on identifying the most pathogenic gingival microorganisms and their molecular targets in host tissues. Currently, over 700 different types of microorganisms living in the oral cavity have been identified [6]. Although protecting tissues from these massively diverse microorganisms seems daunting, animals have evolved to cope with this challenge by using innate and acquired immune systems to counter the potentially harmful invaders. Periodontal tissues are comprised of epithelial and non-epithelial cells that constantly face microorganisms and their byproducts. These cells use specialized innate pattern recognition receptors (PRRs) that recognize ligands called pathogen-associated molecular patterns (PAMPs) [7,8]. Toll-like receptors (TLRs) belong to PRRs that recognize various PAMP molecules produced by dangerous invaders. PAMP-activated TLRSs then signal cells to produce pro-inflammatory cytokines and cellular responses.

Extensive research on various periodontal pathogenic microorganisms such as *Porphyromonas gingivalis* (*P*. *gingivalis*) and *Aggregatibacter actinomycetemcomitans* (*A*. *actinomycetemcomitans*) have brought significant progress to our understanding of their contributions to the pathogenesis of periodontitis [9–19]. Various bacterial molecular components have been identified such as lipopolysaccharide (LPS) and fimbriae that interact with host periodontal tissues and immune systems [20–23]. Bacterial LPS and lipoproteins bind to cell surface PRRs such as TLR2 and TLR4 to activate canonical nuclear factor κ-light-chain-enhancer of activated B cells (NF-κB), cellular signaling that leads to the synthesis and release of various pro-inflammatory cytokines such as IL-1, -6 and TNFα [24–26]. Moreover, recent studies by Nussbaum [27] and Maekawa[13] have recently proposed that *P*. *gingivalis* uses a non-canonical TLR2 signaling pathway to evade host bactericidal activities, providing important insight into the pathogenesis of chronic infection. Sustained inflammation associated with these pathogenic bacteria enhances the activity of osteoclasts, bone-demineralizing cells [28–30]. The development of osteoclasts in the periodontium is due to the increased secretion of the receptor activator of nuclear factor-kappaB ligand, also known as sRANKL, from periodontal ligament cells [31]. Upon binding sRANKL, the receptor (RANK) on pre-osteoclasts in the periodontium causes them to differentiate and demineralize alveolar bones [32–34].

**Fig 1:**
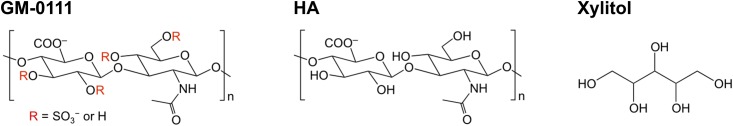
Chemical structures of GM-0111 (A), HA (B), and xylitol (C). GM-0111 shares the same disaccharide backbone with that of HA.

Glycosaminoglycans (GAGs), such as hyaluronic acid, heparin, and more recently synthetic GAGs, have been shown to interfere with bacterial growth, TLR-mediated signaling, and RANKL-induced osteoclast formation [35–41]. GM-0111, a highly sulfated GAG derived from hyaluronic acid (Fig 1), has been shown to reduce inflammation in animal models of bladder pain syndrome (previously known as interstitial cystitis) and rosacea [42,43]. In the present study, we investigated whether GM-0111 disrupts events associated with periodontitis such as the growth and biofilm formation of *P*. *gingivalis*, TLR2/TLR4-mediated cellular activation and osteoclast formation *in vitro*. We report that GM-0111 inhibits multiple events spanning the early pro-inflammatory TLR signaling to molecular pathways activated at the later stage component of bone loss.

## Materials and Methods

### Bacterial culture and scanning electron microscopy

*P*. *gingivalis* strain 2561 (ATCC 33277) was cultured at 37°C in an anaerobic chamber. We used a sterile tryptic soy broth medium containing: 3.0% (w/v) tryptic soy broth, 0.5% (w/v) yeast extract, 0.05% (w/v) _L_-cysteine hydrochloride, 5 μg/mL hemin, and 1 μg/mL vitamin K1. *A*. *actinomycetemcomitans* (ATCC 29523) was also cultured at 37°C in an anaerobic chamber with a few modifications: we used a sterile modified Todd Hewitt broth containing 450 mL of 0.037% Bacto Brain Heart Infusion broth mixed with 10 mL of 12.5% sodium bicarbonate solution, pH adjusted to 7.8. The CO_2_ level was maintained by dissolving Alka-Seltzer (Bayer HealthCare, NJ) in a separate bottle within the culture chamber. Aliquots of an overnight culture of *P*. *gingivalis* and a 3 day culture of *A*. *actinomycetemcomitans* were sub-cultured in fresh medium containing varying concentrations of GM-0111 (detailed method for synthesis appears in reference [44]), HA, or xylitol. Bacterial growth was determined by measuring the absorbance at 600 nm as an approximate measure. We found the absorbance values were unreliable due to the slightly yellow to brown color hues of compounds in culture medium and instead used flow cytometry analysis to count bacteria. These samples were diluted in sterile PBS and sonicated for 5 min in an ultrasonic water bath. Bacteria were then directly counted using preset forward- and side-scatter parameters set to detect *P*. *gingivalis* and *A*. *actinomycetemcomitans* in the culture medium using the Guava HT-8 flow cytometer (EMD Millipore, MA) [45].

To determine the presence of biofilm formation, 100 μL of *P*. *gingivalis* overnight culture was added to 1.5 mL of tryptic broth in sterile 22 mm-glass bottom dishes (Ted Pella #14023–20, Redding, CA). The dishes were gently rocked at 100 rpm for 36 hrs at 37°C in an anaerobic chamber. GM-0111 or HA (final concentration of 0, 0.1, 1, 5, and 10% w/v) was added to the dishes containing bacteria and gently rocked at 100 rpm for an additional 36 hrs at 37°C. The glass bottoms were removed, washed with a 0.15 M sodium cacodylate solution, and incubated in a 0.15 M sodium cacodylate solution containing 2% (w/v) glutaraldehyde, 2% (w/v) paraformaldehyde, and 0.15% (w/v) alcian blue 8GX (dissolved in 3% acetic acid, pH 2.5) in water for 2 hrs at room temperature. These samples were washed 3 times with 0.15 M sodium cacodylate, incubated in a solution containing 0.15 M sodium cacodylate and 1% (v/v) osmium tetroxide for 1 hr at room temperature, and dehydrated in graded ethanol (70%, 20 min; 95%, 20 min; 100%, 30 min). Next, the samples were treated with hexamethyldisilizane for 5 min, desiccated, and sputter coated with gold using a Desk V HP sputterer (Denton Vacuum, Moorestown, NJ) under Ar gas at 30 mA for 30 sec. Samples were examined in blinded fashion using a Quanta 600 FEG scanning electron microscope (Field Emission, Inc., Hillsboro, OR).

### GM-0111 effects on TLR2- and TLR4-mediated cellular responses

The effects of GM-0111 on TLR2- and TLR4-mediated cellular responses were determined using two different cell systems: (1) RAW 264.7 mouse macrophage cells endogenously expressing both receptors along with their signaling complexes and (2) HEK 293 cells heterologously expressing TLR2 or TLR4 along with their respective co-receptors and reporter proteins.

1. RAW 264.7 (ATCC # TIB-71) is a mouse macrophage cell line that expresses all the necessary molecules to activate both TLR2 and TLR4 [46–48]. RAW 264.7 cells were maintained in Dulbeco's modified Eagle medium (DMEM, #SH3028401, GE Healthcare HyClone, UT) supplemented with 50 U/mL penicillin/50 μg/mL streptomycin (#15140–122, ThermoFisher Scientific, NY), and 10% heat inactivated fetal bovine serum (FBSi, #SH30071, GE Healthcare HyClone) in 75-cm^2^ flasks. When the plates reached 50–70% confluency, cells were collected in PBS, washed 3 times in PBS, resuspended in DMEM supplemented with 2% FBSi. Cells were plated in 96-well plates at 3x10^3^ cells/well and cultured overnight. The viability of cells was >85% per experiment. The following day, the cell culture medium was removed, and 180 μL of fresh medium was added to each well. Cells were stimulated with serially diluted Pam3CSK4 (TLR2 agonist, tlrl-pms, InvivoGen) or LPS derived from the *Escherichia coli* (*E*. *coli*) K12 strain (TLR4 agonist, tlrl-peklps). A separate set of plates was prepared with 160 μL of fresh medium, 20 μL of serially diluted GM-0111, and 20 μL of a fixed concentration of Pam3CSK4 or LPS. After culturing for 24 hrs in the presence of ligand, the culture medium samples were collected from each plate and assayed for interleukin-6 (IL-6). The concentrations of IL-6 released from RAW 264.7 cells were measured using Mouse IL-6 ELISA MAX^™^ Deluxe kits (BioLegend, CA).
2. The inhibitory effects of GM-0111 on TLR2 and TLR4 activation were also tested in HEK-Blue^™^ hTLR2 and HEK-Blue^™^ hTLR4 cells (InvivoGen). These cells are HEK293 cell lines stably expressing either human TLR2 or TLR4/MD-2/CD14 along with secreted alkaline phosphatase (SEAP). SEAP is a reporter protein under the transcriptional control by NF-κB and AP-1. Both HEK-Blue hTLR2 and HEK-Blue hTLR4 were maintained in DMEM supplemented with 50 U/mL penicillin, 50 μg/mL streptomycin, 10% FBSi, and HEK-Blue^™^ Selection reagent (InvivoGen) in 75-cm^2^ flasks. Cells grown to 70% confluency were briefly rinsed with PBS and harvested by scraping in 5 mL of PBS. The viability of cells was >85% at the time of harvest. To determine the effects of GM-0111 on TLR2 and TLR4 activation in cells, 20 μL of varying concentrations of GM-0111 were added into 96-well plates. Then, 160 μL of cells resuspended in DMEM supplemented with 10% FBSi were aliquoted into each well. HEK-Blue hTLR2 cells were stimulated with 20 μL of Pam3CSK4, and HEK-Blue hTLR4 cells were stimulated with 20 μL of LPS. The plates were incubated overnight at 37°C. The following day, 5–20 μL of culture medium from each well was tested for TLR activation by measuring SEAP activity using the Quanti-Blue assay reagent.

### Osteoclast culture

The effects of GM-0111 on osteoclast formation were investigated by culturing primary precursor osteoclasts in the presence of varying concentrations of GM-0111. The precursor cells derived from mouse bone marrow were obtained from a commercial source (PMC-OSC13-COS, B-Bridge International, CA) and cultured in a 96-well Osteo Assay plate coated with bone-mimicking, inorganic, crystalline calcium phosphate (Osteo Assay Surface #3989, Corning, MA). Frozen precursor cells were thawed, rinsed with washing medium, and grown in the culture medium supplied by the vendor containing 50 ng/mL of macrophage-colony stimulating factor (M-CSF) and 25 ng/mL of RANKL. Cell culture medium was replaced with fresh medium on day 3 or 4 after starting the culture. GM-0111 was dissolved in PBS at 100 mg/mL and further diluted in the osteoclast culture medium before adding to the cells.

### Measurements of osteoclasts and activities

**Fig 2:**
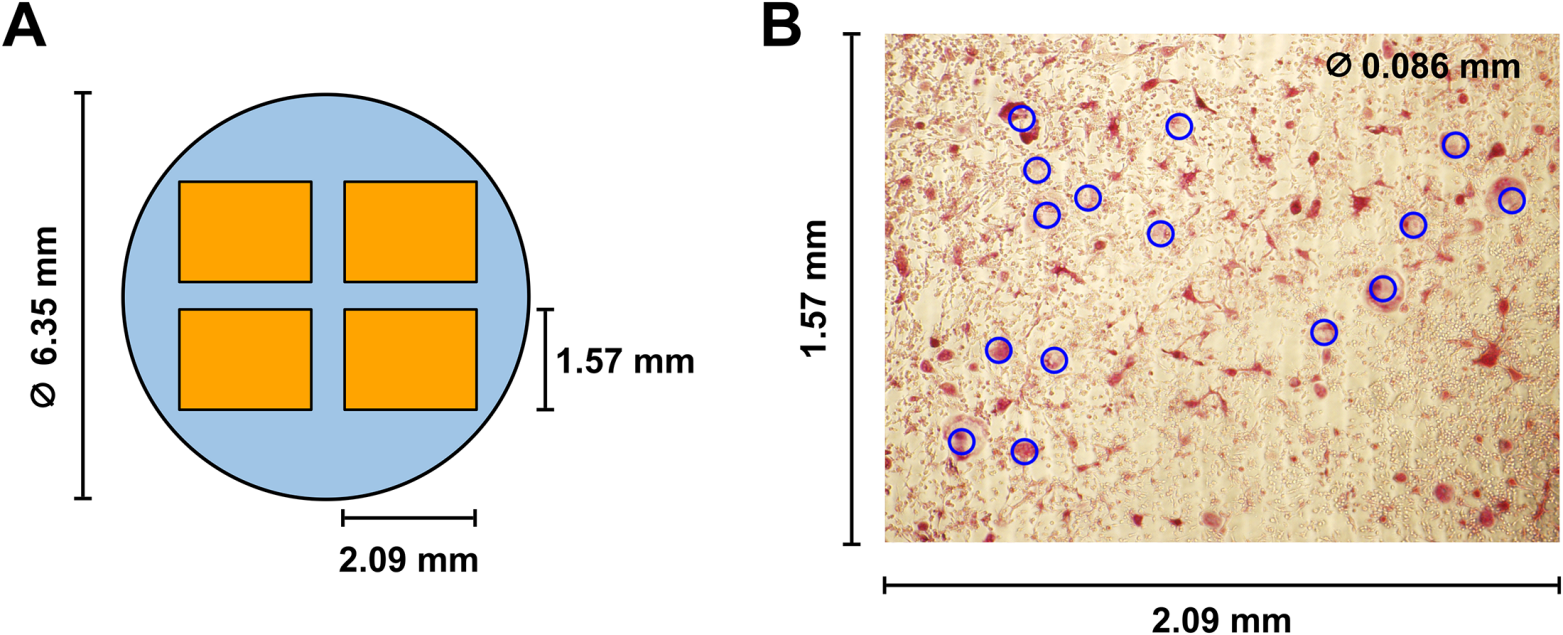
Measurements of multinucleated giant cells (MNGCs) within each microplate well. **A**, Four different rectangular areas (orange) were photographed from each well of the plate. **B**, Cells with diameters of ≥ 0.086 mm (blue circles) were counted as TRAP-positive MNGCs.

Osteoclasts secrete a large amount of tartrate-resistant acid phosphatase (TRAP) and the measurement of TRAP shows osteoclast differentiation and activity [49,50]. To determine the extent of osteoclast formation from precursor osteoclasts, cells in each well were fixed in formalin and stained for TRAP using a commercially available kit (PMC-AK04F-COS, B-Bridge International). Each well was then photographed at four different areas with a frame size of 2.09 mm by 1.57 mm (Fig 2A). Osteoclasts were quantified by counting multinucleated giant cells (or TRAP-positive osteoclasts) with a diameter of ≥86 μm (blue circles, Fig 2B) in each frame. Separate culture wells were treated with a 10% bleach solution to remove cells from the osteoplate. Culture wells were dried, and the resulting pits formed by bone resorbing osteoclasts were photographed for qualitative comparisons. To quantify the effects of GM-0111 on the osteoclast differentiation and activity, we determined the enzymatic activity of TRAP present in cell culture medium. First, 30 μL of cell culture medium from each well was mixed with 170 μL of chromogenic substrate (PMC-AK04F-COS, b-bridge) and incubated for 3 hrs at 37°C. The amount of the resulting enzymatic product was then determined by measuring the absorbance at 540 nm using a microplate reader (Tecan Infinite^®^ F200Pro, Austria).

### GM-0111 binding to RANKL

To test whether GM-0111 inhibits RANKL binding to its receptor, RANK, we first measured GM-0111 binding to RANKL. Heparin binding plates (96-well, cat# 354676, BD Biosciences, MA) were coated with 200 μL of GM-0111 (10 μg/mL) dissolved in phosphate buffered saline (PBS) by incubating overnight at room temperature. The following day, the plate was washed with PBS-T (PBS containing 0.05% Tween-20), blocked with 1% bovine serum albumin (BSA) for 1 hr at 37°C, and then washed. Varying concentrations of recombinant human RANKL (cat# 390-TN, R&D systems, MN) in blocking solution were added to each well and incubated for 2 hrs at 37°C. The plate was then washed with PBS-T, and 50 μL of anti-RANKL antibody solution (0.5 μg/mL; AF626, R&D systems) was added to each well to detect RANKL bound to GM-0111. After 1 hr of incubation at room temperature, the plate was washed with PBS-T and 100 μL of horseradish peroxidase (HRP)-conjugated anti-goat IgG (ThermoScientific, cat# 31402) was added. After 1 hr of incubation at room temperature, the plate was washed with PBS-T, and the bound HRP was determined by incubating with 100 μL of 3,3′,5,5′-tetramethylbenzidine solution (50-77-18, KPL Inc., MD) as a substrate. The enzymatic reaction was stopped with 1 N HCl, and the absorbance at 450 nm was measured using a microplate reader (SpectraMax, Molecular Devices, CA). Binding affinity (K_D_) was calculated from the plot of the absorbance *vs*. the concentration of RANKL.

### GM-0111 effects on RANKL binding to RANK

The effects of GM-0111 on RANKL binding to its receptor RANK were determined by measuring the interaction between RANKL and RANK in the presence of varying concentrations of GM-0111. Polyvinyl 96-well plates were coated with 500 ng/well of recombinant human RANK (cat# 683-RK, R&D systems). Separately, 100 μL of RANKL (500 ng/mL in PBS-T supplemented with 0.1% BSA) was incubated with 100 μL of serially diluted GM-0111 for approximately 16 hrs at 4°C. The RANKL-GM-0111 mixtures were then transferred to the RANK-coated wells and incubated for 1 hr at 37°C. The plates were then washed with PBS-T, and the RANK-bound RANKL was measured following the same steps described for the GM-0111/RANKL binding studies to detect RANKL. The resulting absorbance of RANKL-RANK binding was plotted against the GM-0111 concentration.

### GM-0111 effects on RANKL-mediated cellular response

To determine the effects of GM-0111 on RANKL-mediated cellular responses, we measured NF-κB activation in RAW-Blue^™^ cells (InvivoGen, CA). RAW-Blue cells are RAW 264.7 mouse cells engineered to stably express SEAP reporter protein under transcriptional activation by NF-κB and AP-1. Cells were maintained in 75-cm^2^ flasks (#658170, greiner bio-one, NC) according to the supplier's instructions using DMEM supplemented with 10% FBSi, 50 U/mL penicillin-50 μg/mL streptomycin, and 200 μg/mL Zeocin^™^ (#ant-zn, InvivoGen) as the selection reagent. When the flask reached 50–70% confluency, cells were rinsed once with sterile PBS (#10010–031, Life Technologies, NY), collected in PBS (5 mL/flask) using cell scrapers, and washed with PBS 4 times. The viability of the cells was >90% per experiment. To stimulate cells, 20 μL of serially diluted RANKL solutions were added into 96-well plates, and 180 μL of cell suspension was then added into each well. In separate sets of experiments, 20 μL of serially diluted 10x RANKL solutions and 20 μL of a GM-0111 solution were added into 96-well plates, and 160 μL of cell suspension (5x10^5^ cells/well) was added into each well. Cell suspensions and compound dilutions were made with DMEM supplemented with 5% FBSi. Cells were stimulated with variable concentrations of RANKL for 48 hrs. Aliquots (5–20 μL) of culture medium from each well were tested for SEAP activity using the QUANTI-Blue^™^ (rep-qb, InvivoGen) alkaline phosphatase detection reagent.

### Data Analysis

Biochemical measurements and the number of multinucleated giant cell counts were expressed as the mean and standard of error of the mean (SEM). All measurement data were tested for homogeneity with either Bartlett's test or Fligner Killeen test for equal variances as well as visual inspection of histograms to check the distribution of data [51]. All data sets showed homogeneous distribution supporting the use of parametric tests. The mean differences among treated groups were tested for statistical significance by *one-way* analysis of variance test followed by Dunnet's *t*-test or Tukey's multiple comparison test as *post hoc*. Ligand binding data—RANKL, RANK, and GM-0111—were plotted and fitted by the equation Y = a × [Ligand] + b + (B_max_ × [Ligand])/(K_D_ + [Ligand]) to estimate K_D_ (equilibrium binding constant). Statistical analyses and curve-fits were performed with GraphPad Prism 5.0.4 (GraphPad Software, Inc.) or R (version 3.0.2, The R Foundation for Statistical Computing).

## Results

### Antibacterial effects of GM-0111 on *P*. *gingivalis* and *A*. *actinomycetemcomitans*

**Fig 3:**
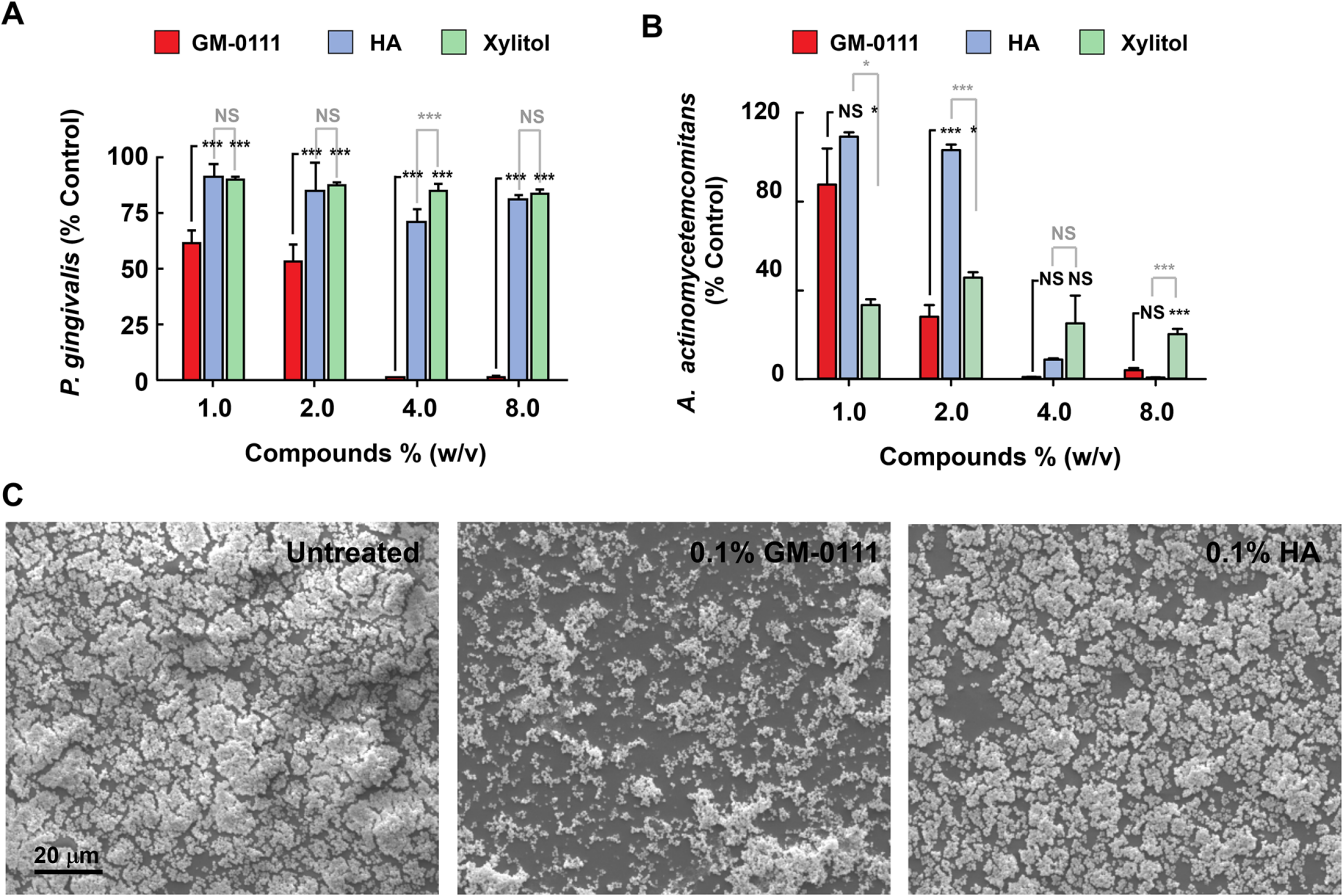
GM-0111 suppresses the growth of *P*. *gingivalis* and *A*. *actinomycetemcomitans* and inhibits the biofilm formation of *P*. *gingivalis*. Flow cytometry-assisted *P*. *gingivalis* (**A**) and *A*. *actinomycetemcomitans* (**B**) counts, grown in the presence of GM-0111, HA, or xylitol show increased growth suppression by GM-0111. **C**, Scanning electron microscopy images of *P*. *gingivalis* cultures grown with GM-0111 (0.1% w/v, middle panel) show reduced biofilm formation compared to control and HA (left and right panels). In **A** and **B**, bars and error bars represent mean ± SEM (n = 4). *p < 0.05, **p < 0.001, NS: not significant (p > 0.05).

To test whether GM-0111 suppresses bacterial growth, we measured the effects of GM-0111 on *P*. *gingivalis* and *A*. *actinomycetemcomitans* growth. At 1–2% (w/v), GM-0111 suppressed *P*. *gingivalis* by 50–60% and *A*. *actinomycetemcomitans* growth by 20–80%, and completely inhibited the growth of both organisms at 4% (w/v) in the medium (Fig 3A and 3B). The antibacterial effects of GM-0111 were compared with HA, the starting material for the synthesis of GM-0111, and xylitol, commonly used in the oral cavity to prevent dental caries [52]. HA or xylitol had negligible effects on *P*. *gingivalis* growth, but did inhibit *A*. *actinomycetemcomitans* like GM-0111 (Fig 3A and 3B). These data suggest that GM-0111 is more effective than HA and xylitol in suppressing the growths of *P*. *gingivalis* and is as effective as HA and xylitol in suppressing *A*. *actinomycetemcomitans* growth.

### GM-0111 suppresses the biofilm formation of *P*. *gingivalis*

To determine whether GM-0111 reduces biofilm formation, *P*. *gingivalis* was cultured as a biofilm on a glass surface in the presence of 0–10% GM-0111 and HA. Biofilm formation was markedly reduced by 0.1% (w/v) GM-0111 (Fig 3C, middle panel), in contrast to 0% GM-0111 and 0.1% HA (Fig 3C, left and right panels). GM-0111 therefore suppresses *P*. *gingivalis* growth both in liquid culture and as a biofilm.

### GM-0111 blocks TLR2- and TLR4-mediated cellular responses

**Fig 4:**
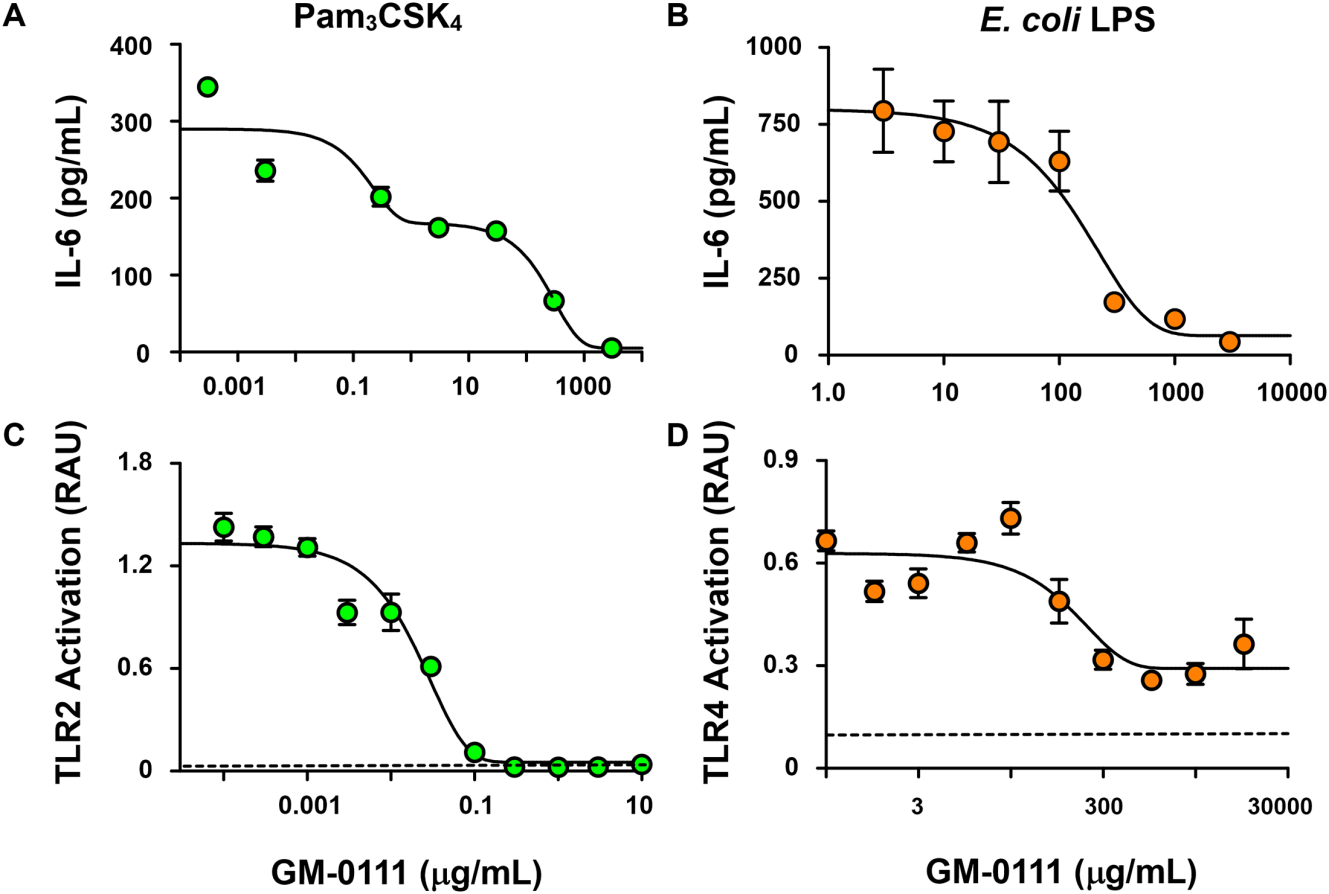
GM-0111 blocks TLR2- and TLR4-mediated NF-κB signaling. Mouse macrophage RAW 264.7 cells secrete IL-6 when stimulated with the TLR2 agonist Pam3CSK4 (1 ng/mL) (**A**) or the TLR4 agonist LPS (1 ng/mL) derived from *E*. *coli* (**B**) (n = 4). GM-0111 blocks these cellular responses by inhibiting TLR2/TLR4-induced NF-κB activation. HEK-Blue hTLR2 (**C**) and HEK-Blue hTLR4 (**D**) cells release reporter protein SEAP (activity measured in Relative Absorbance Unit, RAU) in response to agonist-induced NF-κB activation (n = 5). Symbols and error bars are mean ± SEM. Dotted horizontal lines represent the baseline values of SEAP activities in the culture medium.

To test whether GM-0111 reduces pro-inflammatory cytokine release by inhibiting TLRs, we measured IL-6 release in RAW 264.7 mouse macrophages that endogenously express TLRs. Upon stimulation with the TLR2- or TLR4-specific agonists (Pam3CSK4 or LPS from the *E*. *coli* K strain, respectively), RAW 264.7 cells produced and secreted IL-6 (Fig 4A and 4B). However, the amount of IL-6 released from RAW 264.7 cells was dose-dependently decreased when these cells were treated with GM-0111. These data suggest that GM-0111 inhibits TLR-mediated cytokine release.

To delineate the specificity of GM-0111 on TLR2 and TLR4, we measured TLR2- and TLR4-mediated cellular activation of SEAP reporter protein by NF-κB and AP-1 in HEK-Blue hTLR2 and HEK-Blue hTLR4 cells. Treatment with GM-0111 demonstrated dose-dependent inhibition of TLR2- and TLR4-mediated cellular signaling (Fig 4C and 4D) in HEK-Blue cells. These data are consistent with our RAW 264.7 cell results showing that GM-0111 blocks both TLR2 and TLR4 signaling. In addition, our data indicate that GM-0111 blocks TLR2-mediated signaling at sub-μg/mL concentrations compared to the hundreds of μg/mL inhibitory concentration for TLR4-mediated signaling, illustrating that GM-0111 is a TLR inhibitor with high selectivity to TLR2. Overall, our data demonstrate that GM-0111 inhibits TLR-mediated pro-inflammatory cellular signaling, which is part of the pathogenesis in periodontitis.

### GM-0111 inhibits RANKL-induced osteoclast formation

**Fig 5:**
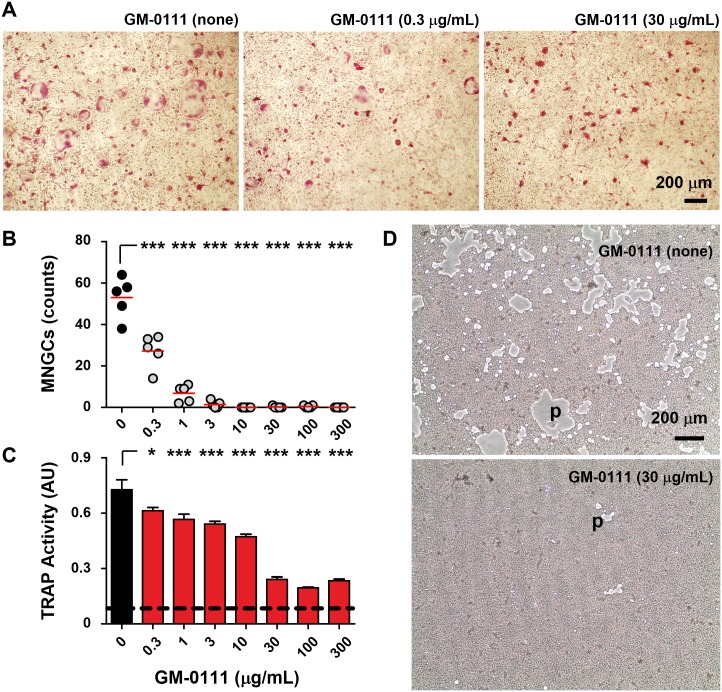
GM-0111 prevents multinucleated giant osteoclast formation. Mouse bone marrow derived pre-osteoclast cells cultured with M-CSF (50 ng/mL) and RANKL (25 ng/mL) transformed into TRAP-positive MNGCs in 4 days (**A**). The number of MNGCs was significantly decreased when pre-osteoclast cells were cultured in the presence of GM-0111 (**A** and **B**). GM-0111 also reduced TRAP secretion from MNGCs (**C**) and the resorption of bone mimetic matrix with the resulting pit formation (empty gray areas, p in **D**). Red lines in **B** represent mean values. In **C**, bars and error bars are mean ± SEM (n = 5) and the dotted horizontal line represent the baseline value of TRAP activity (measured in Absorbance Unit, AU) in culture medium. *p < 0.05 and ***p < 0.001 (compared to the no GM-0111 treated control).

Pre-osteoclasts derived from mouse bone marrow differentiate into TRAP-positive, multi-nucleated giant cells (MNGCs or osteoclasts) within four days when cultured in the presence of M-CSF and recombinant RANKL (rRANKL). When treated with GM-0111, the differentiation of pre-osteoclasts into MNGCs was markedly suppressed (Fig 5A). The anti-osteoclastic effect of GM-0111 was apparent even at 300 ng/mL (Fig 5B). These observations are consistent with our biochemical analysis of TRAP activity in culture medium (Fig 5C), which showed significantly reduced osteoclastic activity in GM-0111-treated cells. In addition, the extent of the bone resorption pits formed by osteoclasts was reduced considerably with GM-0111 treatment (Fig 5D). These results suggest that GM-0111 blocks RANKL-induced osteoclast formation and also reduces consequential bone resorption.

### GM-0111 does not block RANKL-RANK-mediated signaling

**Fig 6:**
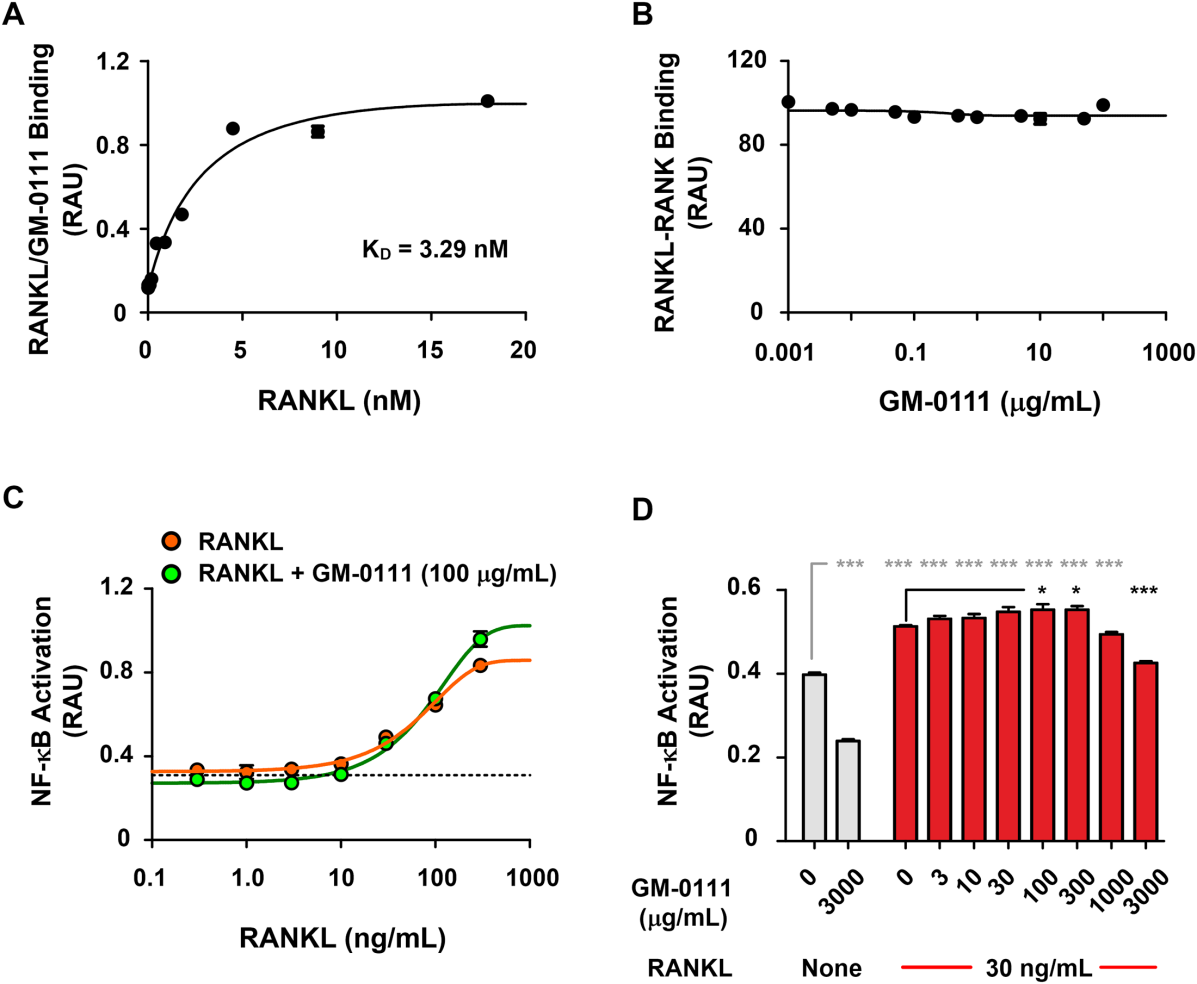
GM-0111 inhibits osteoclast formation independent of RANKL-RANK interaction. GM-0111 binds to RANKL with high affinity (K_D_ = 3.29 nM) (**A**), but does not inhibit the interaction between RANKL and its receptor RANK (**B**) (n = 3). RANKL induces NF-κB-mediated cell signaling in a dose-dependent manner (**C**), but GM-0111 does not reduce RANKL-induced NF-κB signaling (**C** and **D**) (n = 4). Symbols and bars are mean values. Error bars represent SEM. *p < 0.05 and ***p < 0.001 (compared to the controls).

To test the possibility that GM-0111 reduces, RANK activation, we measured the concentration of RANKL bound to a given amount of GM-0111 coated onto 96-well microplates. We found that the interaction between GM-0111 and rRANKL was strong, with a dissociation constant (K_D_) of 3.29 nM (Fig 6A). This binding affinity is similar to monomeric OPG-RANKL binding (~ 4.24 nM), but is approximately 500-fold stronger than RANKL to RANK binding [53].

The high affinity binding of GM-0111 to RANKL may be a major contributing mechanism, interfering with the interaction between RANKL and RANK. To further identify whether GM-0111/RANKL binding can reduce the RANKL-RANK interaction, we first mixed varying concentrations of GM-0111 with rRANKL and added these mixtures to a 96-well microplate coated with rRANK. We then measured GM-0111/rRANKL-bound rRANK. As illustrated in Fig 6B, GM-0111 premixed with rRANKL did not alter the binding properties of rRANKL to rRANK. These results indicate that GM-0111 does not interfere with RANKL-RANK interaction. The absence of blocking effects of GM-0111 on the RANKL-RANK interaction could be a consequence of the recombinant RANK protein lacking transmembrane and intracellular signaling domains present in the native protein. We therefore cannot rule out the possibility that GM-0111 inhibits the RANKL-RANK interaction. We therefore used RAW-Blue cells to monitor the level of downstream SEAP expression to monitor whether GM-0111 inhibits the RANKL-RANK-NF-κB signaling cascade [33]. When stimulated with RANKL, RAW-Blue cells produced and secreted SEAP in a dose-dependent manner as demonstrated in Fig 6C. However, the effects of GM-0111 on RANKL-induced RANK activation were weak, with reduced activation only observable at very high concentrations (3000 μg/mL, Fig 6C and 6D). These data demonstrate that although GM-0111 does bind to RANKL with high affinity, it does not interfere with RANKL-RANK binding or RANKL-induced RANK signaling. Thus, at present, the specific mechanism by which GM-0111 suppresses osteoclast formation remains unclear.

## Discussion

Disrupting the balance between a host and its oral microbiota is a critical element contributing to periodontitis. Therefore, therapeutic strategies to restore host-commensal homeostasis is a logical approach. Current therapy for periodontitis focuses on removing inflammatory sources in the peritoneum by scaling and root planing (SRP), which is often followed by supplemental topical antibiotic treatments to reduce recurring pathogenic bacterial growth in the periodontium. Despite problems of antibiotic resistant microorganisms [54–56], the development of antibiotics such as tetracyclines, quinolones and nitronidazoles continues as an adjunct treatment to SRP [57–60]. Prolonged use of antibiotics can alter the oral microbiome, leading to the establishment of an abnormal microbial ecosystem [61,62]. Strategies to reduce the inflammatory mediators will therefore likely reduce such unintended consequences [25].

Emerging evidence supports the idea that periodontitis could be treated by inhibiting pro-inflammatory molecular signaling pathways such as NF-κB or poly (ADP-ribose) polymerase (PARP) activation, as demonstrated in an animal model of periodontitis [63,64]. GAGs such as HA and synthetic GAGs are anti-inflammatory by inhibiting multiple molecular targets of inflammation. One of these molecular targets include the family of TLRs [65,66]. Bacterial products such as LPS and lipopetides activate TLRs, which induce pro-inflammatory cytokine productions through NF-κB activation [25,67–69]. Our data demonstrate that GM-0111 can inhibit pro-inflammatory cytokine release by blocking TLR2 and TLR4 (Fig 4 and S1 Fig and S1 File). The enhanced expression of TLRs in the periodontium with periodontitis [7,70], as well as significant contribution of TLR2 to alveolar bone loss [71–73] suggest that the inhibitory effects of GM-0111 on TLRs may provide a therapeutic benefit in periodontitis.

Alveolar bone is constantly remodeled by the balanced activities of bone-promoting osteoblasts and bone-resorbing osteoclasts. However, sustained inflammation in the periodontium enhances osteoclastic activity breaking this balance and causing net alveolar bone loss [4,74]. Osteoclasts increase in number and activity, due in part, to the increased production of RANKL by host cells upon prolonged interaction with pathogenic bacteria such as *P*. *gingivalis*. RANKL signals monocytes to differentiate into osteoclasts through RANK-mediated signaling [11,74]. The anti-osteoclastic effects of GM-0111 demonstrated in our studies suggest that GM-0111 has the potential to reduce the alveolar bone loss frequently observed in people with periodontitis.

Our study did not clarify how GM-0111 inhibits the differentiation of pre-osteoclast to bone-resorbing osteoclasts. One possibility is that GM-0111, as well as other types of GAGs, directly binds to RANKL and reduces the activation of RANK. This hypothesis is based on the mechanism of OPG-RANKL interactions, in which free RANKL is directly sequestered by OPG [75,76]. In this scenario, GAGs may function as a scavenger to reduce the amount of free RANKL. Such a possibility has been proposed as a mechanism of heparin to reduce RANKL-induced osteoclast formation [77]. An alternative possibility is that GAG-bound RANKL may block the interaction between RANKL and RANK. Crystal structure studies of OPG bound RANKL suggest that the RANK interacting regions of RANKL become inaccessible upon binding OPG [53,78]. Our data demonstrates that GM-0111 does not inhibit RANKL to RANK binding, which contradicts this scenario. RANKL-induced oligomerization of RANK is a critical step for osteoclastogenesis [74]. It is possible that the GM-0111 may prevent oligomerization of RANKL or inhibit RANKL-induced oligomerization of RANK [74]. It remains difficult to explain the lack of inhibitory effects of GM-0111 on RANKL-induced NF-κB activation in RAW cells.

Recent advances in our understanding of various diseases affecting bone homeostasis provided insights into solving these discrepancies. Although RANKL-RANK signaling is critical for osteoclast formation, cytokines also contribute to osteoclast formation. For example, IL-1β, IL-6, and TNF-α stimulate RANKL production in osteoblasts [79]. GAGs such as heparin reduce the effects of these cytokines [80–82]. GM-0111 shares various biochemical properties of heparin due to its structural similarities and may interact with cytokines affecting osteoclast formation. Furthermore, recent studies indicate that TLRs play important roles in pathogen-mediated osteoclast formation. Activation of both TLR2 and TLR4 receptors are linked to increased expression of RANKL in fibroblast-like synoviocytes in rheumatoid arthritis [83]. Additionally, TLR2 is a necessary component for *P*. *gingivalis*-mediated inflammation and bone loss in mice [84]. Thus, GM-0111 inhibition of TLR2 signaling may provide a clue to the inhibitory effect of this modified GAG on osteoclastogenesis.

Our data suggest that GM-0111 can reduce RANKL-induced osteoclast formation. But what is the source of RANKL and which cells express RANK? More specifically, which cells express high levels of RANKL-RANK that are highly activated by RANKL-RANK interaction? The Crotti and Giannopoulou groups found that the inflammatory cells within the periodontium from periodontitis patients express abnormally higher amounts of RANKL and RANK [85,86]. More specifically, both activated B cells and Th1 lymphocytes were suggested as the major source of RANKL to increase osteoclastogenesis [87]. These cells play important roles in chronic inflammatory lesions. Therefore, prolonged periodontal inflammation consequent to bacterial infection likely contributes to the lymphocytic infiltration and the resulting increased RANKL-RANK promotes osteoclastogenesis.

Biofilm formation by pathogenic bacteria enhances their survival and infection, which contributes to chronic periodontitis. The contributions of different bacterial species in oral microbial ecosystem have been extensively discussed by Socransky and Haffajee [61]. These authors discuss how successive additions of different types of microbial species contribute to periodontitis. For example, an initial colonization of bacteria such as yellow/green/purple microbial complexes create a microenvironment favorable for orange and red microbial complexes that are more pathogenic [61,88]. This sequential colonization model has been regarded as important for designing therapeutic strategies that should focus on creating a microenvironment favorable for less harmful symbiotic microorganisms such as removing dental plaques supplemented with broad spectrum antibiotics.

**Fig 7:**
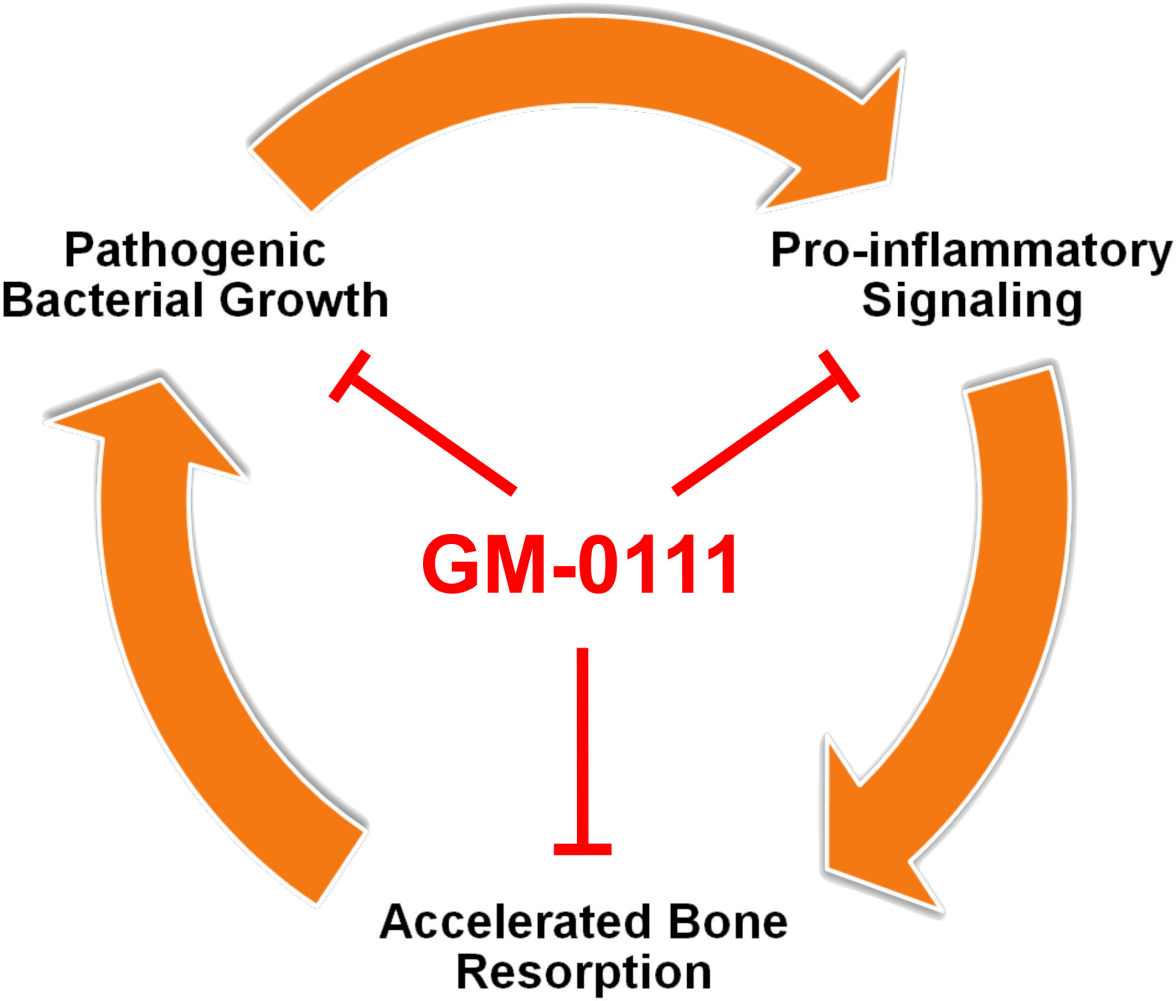
Proposed mechanism of GM-0111 to treat or to prevent periodontitis. GM-0111 (1) reduces the source of inflammatory mediators by suppressing *P*. *gingivalis* growth and biofilm formation; (2) directly blocks pro-inflammatory signaling mediated by TLRs; and (3) also inhibits alveolar bone destruction by preventing osteoclast formation.

In contrast to the sequential colonization model, recent advances in oral microbiology suggest that certain pathogenic microorganisms function as a keystone pathogen [89]. The Hajishengalis group demonstrated that periodontitis could be induced by an extremely low degree of colonization of *P*. *gingivalis* without the successive colonization of other bacterial species in rodents [90]. The keystone pathogen hypothesis provides a new insight into designing therapeutic strategies. In a ligature-induced periodontitis model, vaccination of non-human primates against a specific cysteine protease from *P*. *gingivalis* was effective in reducing periodontitis [91]. These findings suggest that dysbiosis caused by *P*. *gingivalis* could be a mechanism of periodontitis and targeting a set of specific pathogens may provide therapeutic benefit without the risks of using antibiotics. Our data demonstrate that GM-0111 can suppress the growth of *P*. *gingivalis* commonly associated with periodontitis. We also showed that GM-0111 suppresses the growth of *A*. *actinomycetemcomitans* associated mostly with localized aggressive periodontitis [19,92]. When used as a prophylactic, GM-0111 may suppress pathogenic bacterial growth and consequently reduce periodontal inflammation (Fig 7).

## Conclusion

In the present study, we investigated the effects of GM-0111 on molecular events associated with periodontitis, ranging from the anti-inflammatory effects mediated by TLR2 and TLR4, RANKL-induced osteoclast formation, and pathogenic bacterial growth. We propose that the combined effects of GM-0111 on these multiple mediators of periodontitis will be beneficial as a prophylactic or therapeutic for periodontitis. Further studies in animal models of periodontitis will delineate the efficacy as well as the significance of these molecular targets.

## Supporting Information

S1 Fig Flow cytometry analysis showing that GM-0111 does not directly interact with Pam3CSK4 to inhibit TLR2-mediated cell signaling. (A) GM-0111 functionalizes into pLL-coated microbeads. GM-0111CF633 functionalized microbeads were highly fluorescent compared to pLL-coated beads (solid line vs. red histogram). (B) GM-0111-functionalized microbeads were incubated with Pam3CSK4Rhodamine. Histograms show slight increase in fluorescence of GM-0111-functionalized microbeads mixed with 1000 ng/mL of Pam3CSK4Rhodamine. (solid line vs. cyan vs. red histogram). (C) Pam3CSK4Rhodamine. nonspecifically binds to pLL-coated beads. pLL-coated microbeads were mixed with 0 or 1000 ng/mL of Pam3CSK4Rhodamine (solid line vs. red histogram). (D) GM-0111 does not quench Pam3CSK4Rhodamine fluorescence. GM-0111 (without the beads) was mixed with Pam3CSK4Rhodamine and the resulting fluorescence measured. Pam3CSK4Rhodamine fluorescence intensity did not change with GM-0111 (red vs. black bars). Bars are mean and error bars are S.D. values (n = 4).
(TIF)

S1 File Does GM-0111 bind to Pam3CSK4? (DOCX)
