# Supplementary figures and images for: A Modified Glycosaminoglycan, GM-0111, Inhibits Molecular Signaling Involved in Periodontitis
Source: PLoS One. 2016 Jun 16;11(6):e0157310. doi: 10.1371/journal.pone.0157310 (PMC4911086; doi:10.1371/journal.pone.0157310)

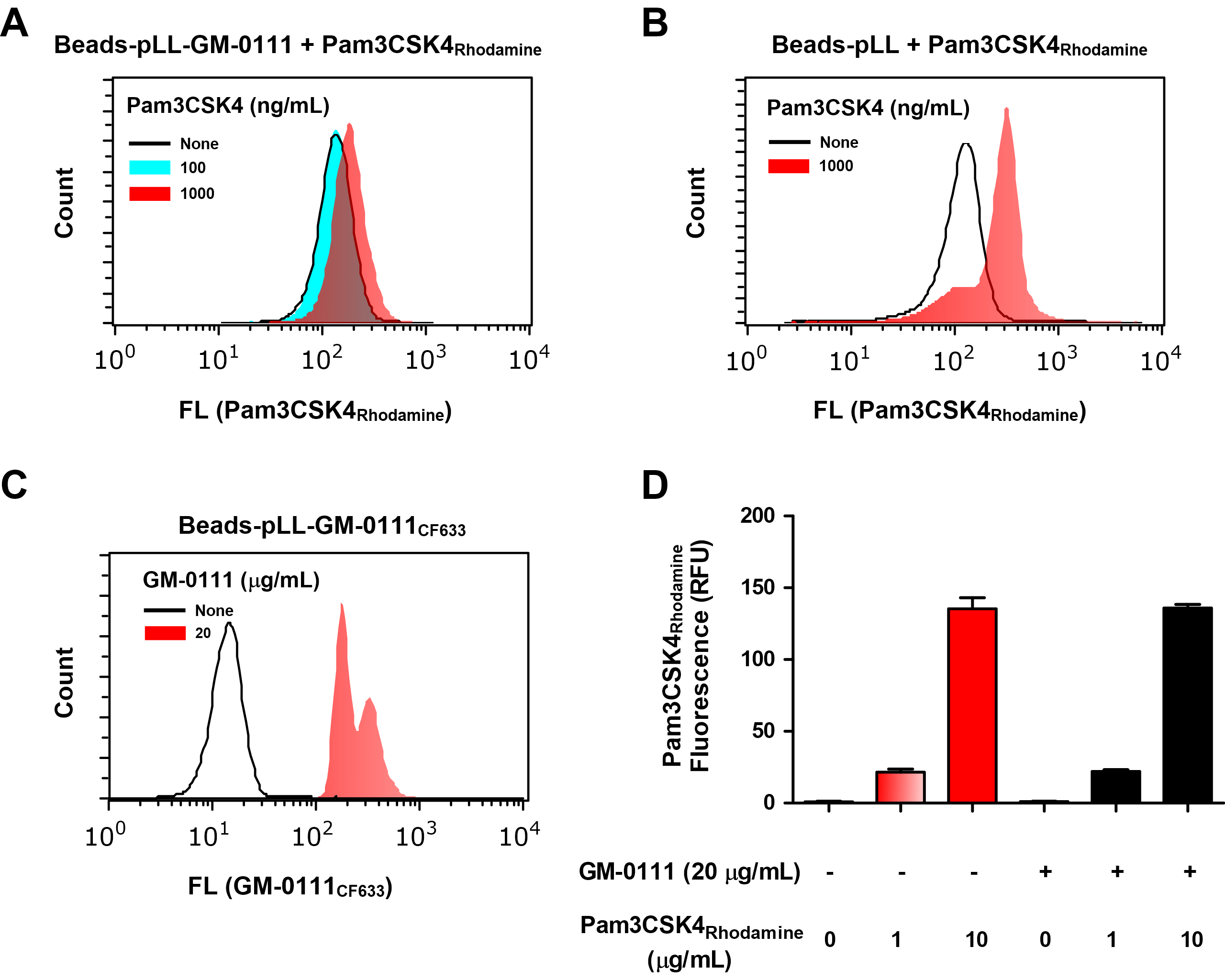

Supplement: S1 Fig — (A) GM-0111 functionalizes into pLL-coated microbeads. GM-0111CF633 functionalized microbeads were highly fluorescent compared to pLL-coated beads (solid line vs. red histogram). (B) GM-0111-functionalized microbeads were incubated with Pam3CSK4Rhodamine. Histograms show slight increase in fluorescence of GM-0111-functionalized microbeads mixed with 1000 ng/mL of Pam3CSK4Rhodamine. (solid line vs. cyan vs. red histogram). (C) Pam3CSK4Rhodamine. nonspecifically binds to pLL-coated beads. pLL-coated microbeads were mixed with 0 or 1000 ng/mL of Pam3CSK4Rhodamine (solid line vs. red histogram). (D) GM-0111 does not quench Pam3CSK4Rhodamine fluorescence. GM-0111 (without the beads) was mixed with Pam3CSK4Rhodamine and the resulting fluorescence measured. Pam3CSK4Rhodamine fluorescence intensity did not change with GM-0111 (red vs. black bars). Bars are mean and error bars are S.D. values (n = 4). (TIF) [file pone.0157310.s001.tif]
