# Supplementary material for: A Modified Glycosaminoglycan, GM-0111, Inhibits Molecular Signaling Involved in Periodontitis
Source: PLoS One. 2016 Jun 16;11(6):e0157310. doi: 10.1371/journal.pone.0157310 (PMC4911086; doi:10.1371/journal.pone.0157310)
Supplement: S1 File — (DOCX) [file pone.0157310.s002.docx]

**Supporting Information S1. Does GM-0111 bind to Pam3CSK4?**

**Materials and Methods**

**CF633 Fluorophore-tagged GM-0111**

GM-0111 was synthesized as previously described [44]. Briefly, 0.4 mL of 1-Ethyl-3-(3-dimethylaminopropyl)carbodiimide (14.0 mg, 0.0731 mmol, 6 eq) and *N*-hydroxysuccinimide (12.6 mg, 0.110 mmol, 9 eq) mixture (dissolved in ultrapure water) was added to 2 mL of GM-0111 solution (67 mg, 0.01220 mmol, 1 eq). The mixture was then stirred for 15 min at room temperature and maintained at pH 4.75 with 3 N NaOH. Then, CF633-hydrazide (#92156, Biotium, Hayward, CA; 2 mg, 0.00189 mmol, 0.2 eq) was added in 0.1 mL of water. The reaction mixture was stirred overnight in the dark at room temperature. The mixture was then dialyzed against 3 L of 100 mM NaCl for 4 hr twice and against 3 L of water for 24 hr twice. The dialyzed solution was then lyophilized to obtain GM-0111_CF633_ as a light blue powder in 75% yield.

**Analysis of GM-0111 binding to Pam3CSK4**

Polystyerene beads (#PP-45-10, Sphereotech, IL) sized around 4.5-4.9 μm in diameter (5% w/v) were prepared as follows. First, 0.5 mL of a microbead solution was washed with equal volumes of phosphate buffered saline (PBS) by centrifugation at 700 x *g* for 5 min then rinsed with 1 mL of water. The washed microbeads pellet was then incubated with 1 mL of 0.01% poly-L-lysine solution for 1 hr in a rocker. Next, microbeads were washed with PBS containing Tween 20 (0.05 % v/v, PBS-T) twice. PLL-coated beads were then mixed 1 mL of GM-0111 solution (20 μg/mL in PBS) for 1 hr at room temp. Microbeads were washed with PBS-T twice to remove unbound GM-0111. GM-0111-PLL-coated microbeads were then incubated with 1% bovine serum albumin (in PBS) overnight at 4°C to block nonspecific binding. Next day, the coated microbeads were collected by centrifugation and incubated with varying concentrations of Rhodamine-tagged Pam3CSK4 (#tlrl-rpms, InvivoGen, CA) for 1 hr at 37°C. The resulting microbeads were then diluted with PBS and immediately analyzed for Rhodamine fluorescence using Guava 8-HT flow cytometer. Possible quenching effects of GM-0111 on Rhodmaine fluorescence was tested by measuring the fluorescence of solutions containing 20 μg/mL of GM-0111 with varying concentrations (1 and 10 ng/mL) of Pam3CSK4-Rhodamine. Rhodamine fluorescence in the solution was measured after a 2 hr incubation at 37°C with a microplate reader (Excitation λ_530 nm_ and Emission λ_580 nm_).

**Results**

To determine whether GM-0111 inhibits TLR2-mediated cell signaling by sequestering agonists, we measured the binding of GM-0111 to Pam3CSK4. Polystyrene microbeads were first coated with poly-L-lysine (pLL) and then functionalized with fluorescently labeled GM-0111 (GM-0111_CF633_). S1(A) **Fig.**  demonstrates efficient functionalization with GM-0111_CF633_ as detected by flow cytometry. In a second experiment, we functionalized PLL-coated microbeads with non-fluorescent GM-0111, and incubated the microbeads in solution with Rhodamine-tagged Pam3CSK4 (Pam3CSK4_Rhodamine_) to detect its binding to GM-0111. If GM-0111 directly binds to Pam3CSK4, these particles will be fluorescent. However, GM-0111 functionalized microbeads mixed with 100 ng/mL Pam3CSK4_Rhodamine_ did not show any fluorescence, and there was only a minimal increase of fluorescence in microbeads mixed with 1000 ng/mL of Pam3CSK4-Rhodamine (**S1(B) Fig.**). By contrast, pLL-coated beads mixed with Pam3CSK4_Rhodamine_ showed intense fluorescence suggesting that Pam3CSK4_Rhodamine_ could also bind to pLL-coated beads (**S1(C) Fig.**). Thus, we measured the fluorescence of Pam3CSK4_Rhodamine_ mixed with GM-0111 (without the beads) to test whether the low fluorescence of GM-0111/ Pam3CSK4-Rhodamine was quenched. The fluorescence of Pam3CSK4_Rhodamine_, however, was unaffected by added GM-0111, suggesting that GM-0111 might not directly interact with Pam3CSK4. Overall, we have no clear evidence that GM-0111 inhibits TLR2 cell signaling by sequestering or neutralizing its agonist.

**S1 Figure**. **Flow cytometry analysis showing that GM-0111 does not directly interact with Pam3CSK4 to inhibit TLR2-mediated cell signaling.** (A) GM-0111 functionalizes into pLL-coated microbeads. GM-0111_CF633_ functionalized microbeads were highly fluorescent compared to pLL-coated beads (solid line *vs*. red histogram). (B) GM-0111-functionalized microbeads were incubated with Pam3CSK4_Rhodamine_. Histograms show slight increase in fluorescence of GM-0111-functionalized microbeads mixed with 1000 ng/mL of Pam3CSK4_Rhodamine_. (solid line *vs*. cyan *vs*. red histogram). (C) Pam3CSK4_Rhodamine_. nonspecifically binds to pLL-coated beads. pLL-coated microbeads were mixed with 0 or 1000 ng/mL of Pam3CSK4_Rhodamine_ (solid line *vs*. red histogram). (D) GM-0111 does not quench Pam3CSK4_Rhodamine_ fluorescence. GM-0111 (without the beads) was mixed with Pam3CSK4_Rhodamine_. and the resulting fluorescence measured. Pam3CSK4_Rhodamine_ fluorescence intensity did not change with GM-0111 (red *vs*. black bars). Bars are mean and error bars are S.D. values (n = 4).
